# Clinical Presentation and Magnetic Resonance Findings in Sellar Tuberculomas

**DOI:** 10.1155/2014/961913

**Published:** 2014-07-09

**Authors:** Dulce Bonifacio-Delgadillo, Yolanda Aburto-Murrieta, Citlaltepetl Salinas-Lara, Julio Sotelo, Ivonne Montes-Mojarro, Arturo Garcia-Marquez

**Affiliations:** ^1^Hospital Angeles Xalapa, Carretera México-Veracruz No. 560, Esq. Camino a Pastoresa, Colonia Pastoresa, CP 91193, Mexico; ^2^Instituto Nacional de Neurología y Neurocirugía Manuel Velasco Suárez, Insurgentes sur 3877, Colonia la Fama, 14269 Mexico, DF, Mexico

## Abstract

*Background and Importance*. Sellar tuberculomas are extremely rare lesions with nonspecific clinical manifestations. The tuberculous infection of the pituitary gland and sellar region is characterized by the presence of an acute or chronic inflammatory reaction and may occur in the absence of systemic tuberculosis. The diagnosis is difficult prior to the surgery. An adequate diagnostic and antituberculous drugs usually result in a good outcome. *Clinical Presentation*. We report four cases of sellar tuberculoma, 3/1 female/male, age range: 50–57 years. All patients had visual disturbances and low levels of cortisol. *Conclusion*. The clinical diagnosis of sellar tuberculoma is a challenge and should be suspected when a sellar lesion shows abnormal enhancement pattern and stalk involvement, and absence of signal suppression in FLAIR.

## 1. Introduction

Tuberculosis is the second most common cause of death from infectious diseases after HIV/AIDS; it is also the leading cause of death from an infectious disease in HIV coinfected patients [1]. Tuberculoma of the central nervous system can occur at any site in any age group [2]. They represent 0.15%–4% of intracranial space occupying lesions [3]; their most frequent location are the cerebrum and cerebellum, less frequent are the brain stem, basal ganglia, and thalamus. However, the sellar localization of a tuberculoma is extremely rare even in postmortem examination. They may occur in the absence of systemic tuberculosis. Tuberculomas of the sellar and suprasellar region comprise 1% of all intracranial tuberculomas; [2] a review of the literature reveals only 67 cases reported (Table 1) [2–37]. This entity represents a diagnostic challenge that needs to be solved by clinical, imaging, and histological analysis. Histological examination shows granulomas with central caseous necrosis. The differential diagnoses of intrasellar lesions include: adenoma, cyst of Rathke's pouch, craniopharyngioma, glioma of the optic chiasm or hypothalamus, meningioma, germ cell tumor, hamartoma, lipoma, dermoid or epidermoid cyst, metastasis; and granulomatous entities like lymphocytic hypophysitis, sarcoidosis, or Langerhans' histiocytosis [37]. Pituitary tuberculoma should be considered in the differential diagnosis of sellar masses because in most cases of pituitary tuberculosis antibiotic treatment is effective [1].

This report highlights some radiological clues in four cases of sellar tuberculomas to suspect the diagnosis and reviews the literature.

## 2. Case Presentation

### 2.1. Case  1

**Figure 1:**
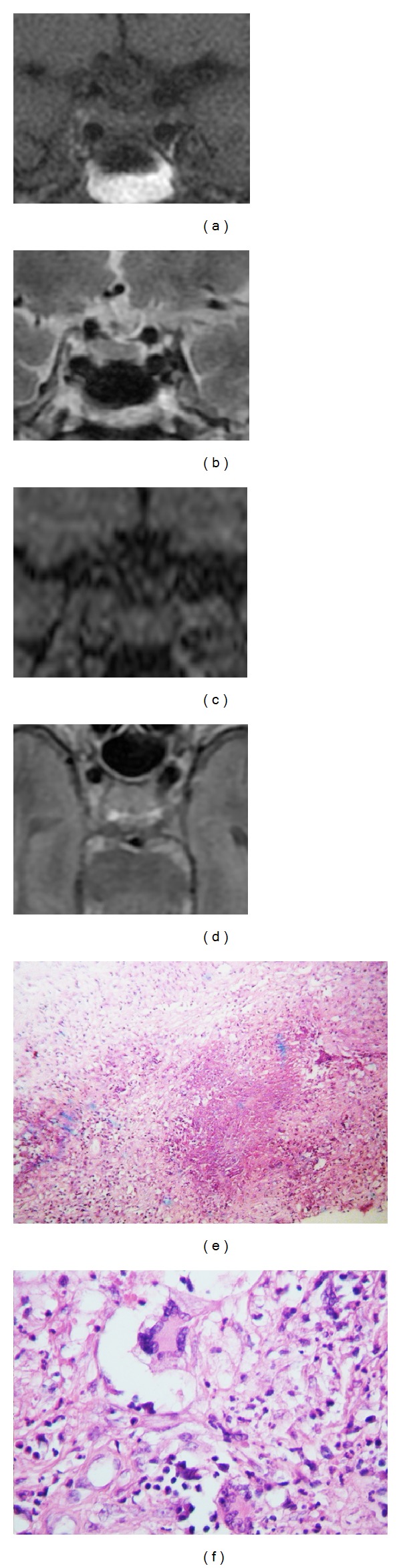
MRI T1-weighted (a), T2-weighted (b), and contrast enhanced T1-weighted (c) of a 67-year-old woman presenting with impaired vision and temporal visual field deficit two months before admission. MRI was done two months after the surgery with pterional approach and showed persistence of intrasellar lesion with stalk involvement. Photomicrograph ×40 H & E (e) and photomicrograph ×400 H & E (f) show central caseous necrosis with lymphocytic inflammatory infiltrate, extensive fibrosis, Langhans multinucleated giant cell with epithelioid cells, and lymphocytes, with few polymorphonuclear.

A 67-year-old woman presented with impaired vision and temporal visual field deficit two months before admission. General physical examination was normal. She was alert, the visual acuity in the right eye was 20/200, in the left was limited to eye-counting fingers because of cataract, color vision 0/8 bilateral, optic nerve bilateral pallor, bilateral islands of nasal vision, and endocrine investigation disclosed low levels of cortisol. CT scan of the pituitary region showed an intrasellar heterogeneous mass with peripheral enhancement. Preoperative MRI was not available. Prior to surgery the diagnosis was pituitary adenoma versus inflammatory disease. The patient underwent pterional surgery of the pituitary region. Histology revealed central caseous necrosis with lymphocytic inflammatory infiltrate, extensive fibrosis, and Langhans multinucleated giant cells with epithelioid cells. An MRI two months later showed persistence of intra- and suprasellar inflammatory lesion that reached the chiasm and involved the stalk (Figure 1). Diagnosis of sellar tuberculoma was made; the patient received isoniazid, rifampin, pyrazinamide, ethambutol, and steroids during eight months. Eighteen months later, MRI showed resolution of the lesion.

### 2.2. Case  2

**Figure 2:**
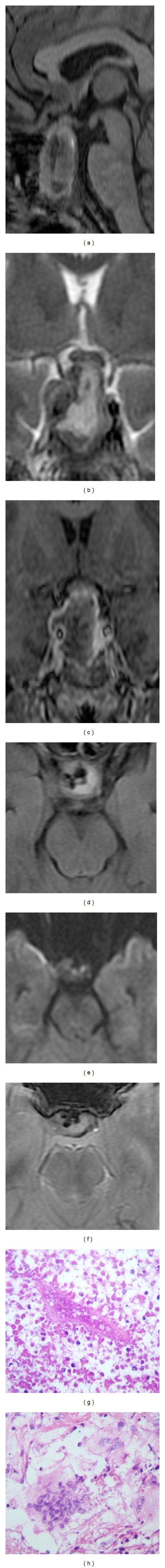
(a) MRI sagittal T1-weighted, (b) coronal T2-weighted images, (c) coronal T1-weighted images after contrast media administration, (d) axial T2-FLAIR-weighted images, (e) axial diffusion weighted images, and (f) T2∗-weighted images of a 50-year-old woman with headache associated with raised pressure and vertical diplopia revealed a sellar and suprasellar lesion with hemorrhagic areas displacing dorsally the chiasm, thickening the infundibulum and cavernous sinus extension predominantly to the right side. (g) Photomicrograph ×100 H & E and (h) photomicrograph ×400 H & E showed pituitary parenchymal necrosis with blood vessel and group of multinucleated giant cells surrounded by lymphocytes and epithelioid cells.

A 50-year-old woman had a sudden and intense throbbing headache associated with raised pressure and vertical diplopia. On admission she was alert, visual acuity in the left eye was 20/80, in the right eye it was 20/200, and she had palsy of right third and fourth nerves. Color vision, optic nerves, and visual fields were normal. Endocrine investigation disclosed low contents of cortisol in serum. MRI images revealed a sellar and suprasellar lesion displacing dorsally the chiasm, thickening of the infundibulum, and cavernous sinus extension, predominantly to the right side (Figure 2). The diagnosis was inflammatory lesion of hypofisis. A transsphenoidal biopsy was obtained. Histopathology reported pituitary parenchymal necrosis with blood vessels surrounded by multinucleated giant cells, lymphocytes, and epithelioid cells; Ziehl Neelsen staining was positive and the culture was positive to* Mycobacterium *spp. The patient received isoniazid, rifampin, pyrazinamide, and streptomycin during one year. Follow-up MRI revealed resolution of sellar tuberculoma.

### 2.3. Case  3

**Figure 3:**
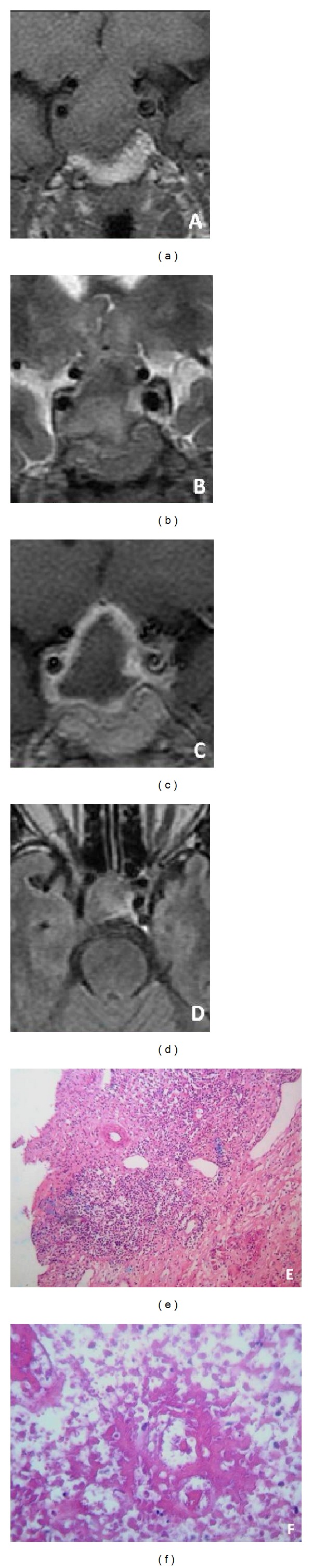
(a) Coronal T1-weighted images, (b) coronal T2-weighted images, and (c) coronal T1-weighted images after contrast media administration views of initial MRI of a 59-year-old man presented with of fever, nausea, vomiting, polyuria, and loss visual acuity. Intra- and suprasellar lesion contacting bilaterally the gyrus rectus and the chiasm. (e) Photomicrograph ×100 H & E and (f) photomicrograph ×100 H & E showed extensive fibrosis with lymphocytic infiltration and areas of trapped pituitary cells, vasculitis with blood vessel necrosis, adjacent tissue, and few pituitary cells.

A 59-year-old man had acute onset of fever, nausea, vomiting, polyuria, and loss of visual acuity for 8 days. On admission he was febrile. General examination was normal. At neurological exam he was confused and somnolent, with incoherent speech; blood examination showed hyponatremia. Visual acuity in the left eye it was 20/100, in the right eye was 20/200, left optic nerve was edematous, and there was palsy of right third nerve. Color vision and visual fields were normal. Endocrine investigation disclosed significant deficiency of the thyrotrophic and corticotropic hormones in serum. The MRI showed an intra- and suprasellar inflammatory lesion contacting bilaterally the gyrus rectus and the chiasm (Figure 3). The clinical diagnosis was sellar tumor, transsphenoidal biopsy was done, and histopathology revealed extensive fibrosis with lymphocytic infiltration in areas of trapped pituitary cells, vasculitis with blood vessel necrosis, and filiform Ziehl Neelsen positive structures. The patient received antituberculosis treatment in another institution for pulmonary tuberculosis. Twenty-four months later cranial tomography did not reveal sellar lesion.

### 2.4. Case  4

**Figure 4:**
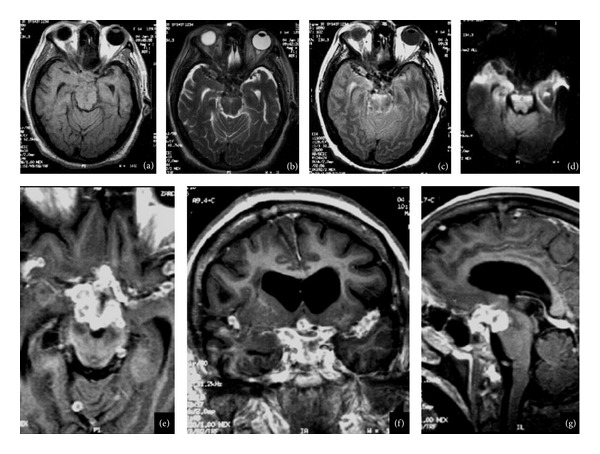
(a) Axial T1-weighted images, (b) axial T2-weighted images, and (c) axial T2- and T2-FLAIR-weighted images after contrast media administration. (e) Axial plane, (f) coronal, (g) sagittal MRI images of 57-year-old woman, six years before she had tuberculous meningitis, obstructive hydrocephalus secondary to multiple coalescent nodular well-defined images; the biggest was localized in the sella, the lesion in contact with the chiasm, and extends to cavernous sinuses. Multiple lesions were localized in basal cisterns and both lateral fissures and the retrosellar extension of the lesions cause brain stem compression.

A 57-year-old woman, six years before she had tuberculous meningitis documented by PCR and MRI, was treated with steroids and ethambutol during seven months. Seven days before admission she had throbbing headache associated with fever and vomiting. On admission she was febrile and confused with right ptosis, mydriasis, left hemiparesis, and meningism. Visual acuity was not recorded and endocrinology profile was not done. MRI images (Figure 4) shows obstructive hydrocephalus secondary to multiple coalescent well defined nodules, the largest was localized in the sella and involve the chiasm and cavernous sinus; the lesions localized in basal cisterns and retro sellar region cause brain stem compression. Meningitis and sellar tuberculosis were diagnosed based on the medical history, radiologic findings, and PCR to tuberculous bacilli in serum samples. She was discharged with antituberculous treatment; follow-up studies were not available.

## 3. Results

Age range of patients was 50–67 years, mean: 58 years; male/female ratio was 1 : 3. The predominant clinical features were headache and impaired visual acuity; clinical results are summarized on Table 1.

According to the extension three types of lesion were seen.

Two patients presented sellar and suprasellar masses without obstructive hydrocephalus, both patients presented visual impairment: one with bilateral optic atrophy and the other with papilloedema. Systemic manifestations of tuberculosis were not found. One patient had retrosellar extension, brain stem compression, and hydrocephalus with raised pressure manifestations (right third nerve palsy and right upper motor neuron); he also had pachymeningitis with meningism. One patient presented severe headache and palsy of right third and fourth nerves and decreased visual acuity.

## 4. Discussion

Tuberculoma is defined as a well-circumscribed mass composed of granulomatous chronic inflammatory tissue that may occur in the cerebral hemispheres, cerebellum, brain stem, or perimeningeal spaces [38]. They account for 0.15% to 4% of intracranial space-occupying lesions with geographical variations [3]. In India, sellar and suprasellar tuberculomas represent 1% of all intracranial tuberculomas [2]. We found 27 cases of intracranial tuberculomas in the registers of the* National Institute of Neurology and Neurosurgery of Mexico* since 1999 to 2009, the localization was sellar in four of them (14%) higher than reported in similar studies (Table 1). The pathway for spreading mycobacterium tuberculosis to this region is unclear; either haematogenous spreading or contiguous extension from a local tuberculous infection of the paranasal sinuses has been proposed [39]. In some patients the primary focus cannot be demonstrated. From the cases reported here, one patient had antecedent of tuberculous meningitis and another had pulmonary infection. Headache is a common symptom; throbbing severe headache was present in 2 patients. All patients had visual disturbances as affection of visual fields, visual acuity, diplopia, mydriasis, or blurred vision, in contrast with other reports in which visual disturbances were present only in about 50% of cases. One patient had fever, meningism, and Weber syndrome at onset due to a mass effect on the brain stem; this syndrome had not been previously reported in cases of sellar tuberculoma.

Similar reports found anterior pituitary hormone deficiencies or central diabetes insipidus at onset, hypogonadism secondary to low levels of GH, FSH/LH, TSH, and ACTH deficiencies [40]. All our cases with endocrine system investigation (*N* = 3) disclosed a decrease of cortisol levels; one patient had deficiency of the thyrotrophic hormone. Endocrinology profile was not made in one patient. A previous review revealed that 60% of patients had complete anterior panhypopituitarism and 28% had central diabetes insipidus as initial presentation [41]. Another frequent finding is raised prolactin levels due to pituitary stalk effect [33].

Pituitary tuberculosis may present a diagnostic dilemma because it is difficult to differentiate inflammatory lesions from a pituitary adenoma. MRI is the chosen imaging modality to establish the differential diagnosis. Thickening and nodularity of the pituitary stalk are considered to be a sign of pituitary tuberculoma; however, this finding is nonspecific as it is also seen in other inflammatory conditions like sarcoidosis, syphilis, and idiopathic hypophysitis [12, 42, 43]. This finding was present only in one patient (Case  2) (Figure 2(b)). The classical imaging of brain tuberculomas is multiple coalescing contrast-enhancing lesions [44]. We also found this pattern only in one patient (Case  4). T1-weighted image usually are hypointense; nevertheless, they can also be hyperintense too due a high protein content [2]. Three of four cases were isointense on T1-weighted image. The literature described that the lesions may appear hyperintense on T2-weighted image or may have an hyperintense center surrounded by a hypointense rim with peripheral ring enhancement of the lesion and enhancement of the adjacent dura and basal cistern. Nonenhancement areas corresponded to tuberculoma caseation [2]. On T2-weighted images we observed a major hyperintense component in three cases (75%), similar to other reports; we found peripheral ring enhancement in three cases (75%), whereas in one case generalized slightly enhancement was seen. On T2-FLAIR images all lesions were hyperintense; this finding could be useful for the differential diagnosis with other inflammatory lesions.

The histopathological characteristic of tuberculosis is ill-defined caseating granulomas with giant cells. Other causes of granulomatous inflammation of the pituitary are lymphocytic hypophysitis, sarcoidosis, and Langherhan's histiocytosis and caseating giant cell granulomata [2]. The diagnosis requires confirmation by acid fast Bacilli and chain polymerase reaction for detection of mycobacterial DNA; however, these studies are not routinely done [27]. In our cases the histological studies revealed chronic inflammatory lesions of granulomatous type in different phases, leading to different degrees of destruction and necrosis of the pituitary gland.

The presence of vasculitis is a serious consequence of tuberculous meningitis, which can result in infarction [45]. This finding was evident in one of our cases. Endocrinological study and Ziehl Neelsen staining together with imaging and histology can confirm the diagnosis. In one patient, the culture of brain biopsy sample on Lowenstein-Jensen medium was required to confirm the diagnosis.

Antituberculous therapy is mandatory in sellar tuberculosis; most cases have appropriate response. One patient could not be followed. Three cases had resolution of pituitary tuberculoma in follow-up studies (12–24 months). There is no consensus regarding the type of antitubercular regimen and duration of the treatment in pituitary tuberculoma.

## 5. Conclusion

In the present study this entity was more frequent than previously reported, so it should be taken into account in the differential diagnosis of sellar lesions. Clinically, it behaves like any sellar lesion with visual manifestations and endocrine alteration. Sellar tuberculoma should be suspected when a sellar lesion shows abnormal enhancement pattern and stalk involvement; peripheral enhancement and absence of signal suppression in FLAIR are clues to the imaging diagnosis. Long-term chemotherapy with antituberculous drugs usually results in a good outcome.

## Figures and Tables

**Table 1:** Series previously reported.

Author, year (reference number)	Patient Sex/Age	Clinical presentation
Garlan and Armitage, 1933 [4]	2 patients	Age and gender not mentioned in the paper
Coleman and Meredith, 1940 [5]	1 patient	Primary optic atrophy and bitemporal hemianopia
Glass and Davis, 1944 [46]	M/54 Y	Panhypopituitarism with febrile episodes
Brooks et al., 1973 [6]	F/33 Y	Headache, diminution of vision, and past history of pulmonary Koch
Eckland et al., 1987 [7]	F/37 Y	Bitemporal headache, vomiting, and diplopia. Right sixth nerve palsy, right temporal hemianopia and a depressed right corneal reflex lateral gaze to the right
Esposito et al., 1987 [8]	F/54 Y	Headache with loss visual acuity in the left eye and diplopia on left lateral gaze. History pulmonary tuberculosis at the age of 22
Delsedime et al., 1988 [9]	F/45 Y	Isolated tuberculous granuloma of the hypophysis with no other systemic localizations
Kamiya et al., 1991 [10]	M/41	Headache, history of pulmonary tuberculosis at the age of 20
Taparia et al., 1992 [34]	M/40 Y	Intermittent dull headache. Visual acuity reduced and bilateral optic atrophy
Ghosh and Chandy, 1992 [11]	F/35 Y	Headache, vomiting, blurred vision, amenorrhea, and galactorrhea
Ranjan and Chandy, 1994 [36]	Five patients	In four cases the clinical and radiological diagnosis was that of a pituitary adenoma. One patient presented similar to a subarachnoid haemorrhage, but the CSF analysis was suggestive of tuberculous meningitis. All these patients presented either with intermittent headache. Hypopituitarism was diagnosed in two patients and one patient had an associated galactorrhoea-amenorrhoea syndrome. Only one patient had a bitemporal field cut. In all other patients ophthalmological examination was normal
Pereira et al., 1995 [12]	F/55 Y	Left sixth nerve palsy and headaches
Ashkan et al., 1997 [13]	F/33 Y F/31 Y	Secondary amenorrhea, fatigue, headache and weight loss Secondary amenorrhea, galactorrhea and headache
Petrossians et al., 1998 [35]	F/54 Y	Extreme weakness, headache, and vomiting
Gazioğlu et al., 1999 [14]	F/34 Y	Acromegaly, oligomenorrhea, and hypertrichosis
Sharma et al., 2000 [15]	18 cases Range: 8–43 Y	The duration of symptoms varied from 15 days to 2 years (average 4 months); the most common symptoms being headache followed by decrease or loss of vision. Five patients had features of panhypopituitarism whereas three had raised prolactin (PRL) levels. In three patients, there was past history of pulmonary tuberculosis
Basaria et al., 2000 [16]	F	Pituitary mass, presumed preoperatively to be an adenoma. The patient did not have history of tuberculosis infection
Arunkumar and Rajshekhar, 2001 [3]	M/27 Y	3 previous episodes of pituitary apoplexy
Kumar et al., 2001 [17]	1 patient	Pituitary mass with clinical and MRI findings consistent with adenoma
Manghani et al., 2001 [37]	F/24 Y	Headache and loss of libido
Domingues et al., 2002 [18]	F/46 Y	Confusion and hypopituitarism with no evidence of systemic tuberculosis
Stalldecker et al., 2002 [19]	F/16 Y	Headache, hyperpyrexia, polyuria, polydipsia and amenorrhea
Desai et al., 2003 [20]	F/15 Y F/19 Y F/22 Y F/30 Y M/47 Y	Headache, amenorrhoea, galactorrhoea, diminution of vision, bitemporal hemianopia, past history of pulmonary Koch Headache, amenorrhoea Headache, amenorrhoea, diminution of vision, bitemporal hemianopia, past history of Koch's cervical adenopathy Headache, oligomenorrhoea, galactorrhoea Headache
Satyarthee and Mahapatra, 2003 [21]	F/32 Y	Diabetes insipidus and secondary amenorrhea
Harzallah et al., 2004 [22]	F/52 Y M/62 Y	Extreme weakness, headache, vomiting, meningeal syndrome and third cranial nerve palsy Generalized tonic-clonic seizure, low grade fever and mental confusion
Trabelsi et al., 2005 [23]	F/42 Y	History of erythema nodosum, poliuria, polydipsia, amenorrhea and galactorrhea
Deogaonkar et al., 2006 [24]	F/27	Headaches, left ptosis and left facial numbness. Drowsy and obtunded, tachycardia with blood pressure normal. Left facial hypoesthesia in left V1 and V2 distribution. Her hormone profile did not reveal any abnormality
Bayindir et al., 2006 [25]	1 patient	Age and gender no mentioned in the article
Sunil et al., 2007 [2]	M/42 Y	Polyuria, polydypsia, polyphagia, and decreased libido secondary to diabetes mellitus
Yilmazlar et al., 2007 [26]	F/37 Y	Galactorrhea and menstrual irregularity
Husain et al., 2008 [27]	F/40 Y	Headache and fatigue
Rao et al., 2008 [28]	F/47 Y	Diabetic, hypothyroid and hypertensive. Presented with headache, vomiting, transient blurring of vision and galactorrhea.
Behari et al., 2009 [29]	8 cases, Range: 15–40 Y M : F ratio = 5 : 3	Range of duration of clinical symptomatology, 6 months–3 years Headache was again the predominant symptom in most patients, which resulted from raised intracranial pressure due to both the large size of the lesion as well as the coexisting hydrocephalus. One patient presented with headache due to pachymeningitis, one due to stretching of the diaphragma sellae by an intrasellar tuberculous abscess, and the third due to clival infiltration. Three of our patients had either a previous history of tuberculosis or a concomitant lesion at some other site
Mittal et al., 2010 [30]	F/40 Y	Persistent headache and blurred vision on the left side
Domiciano et al., 2010 [31]	F/33 Y	Nodular RA who was being treated with methotrexate, sulfasalazine and corticosteroids and presented with subcutaneous nodules simultaneously with aseptic meningitis. Mycobacterium tuberculosis was identified in cultures from a biopsy of an axillary nodule. The patient also developed polyuria and polydipsia with normal glycemia
Shukla et al., 2010 [32]	M/68 Y	Holocranial headache of four months duration with left temporal hemianopia, with visual acuity of 6/6, without any localizing sign
Furtado et al., 2011 [33]	F/31 Y	Panhypopituitarism
